# Pesticide Use and Risk Perceptions among Small-Scale Farmers in Anqiu County, China

**DOI:** 10.3390/ijerph14010029

**Published:** 2016-12-30

**Authors:** Jianjun Jin, Wenyu Wang, Rui He, Haozhou Gong

**Affiliations:** College of Resources Science and Technology, Beijing Normal University, Beijing 100875, China; 201521190014@mail.bnu.edu.cn (W.W.); 201621190023@mail.bnu.edu.cn (R.H.); hzgong@163.com (H.G.)

**Keywords:** pesticide use, risk perception, pesticide overuse, small-scale farmer, China

## Abstract

The unsafe use and misuse of pesticides in China are major threats to farmers’ health and the environment. The purpose of this study is to evaluate small-scale farmers’ practices with regard to pesticide use and identify the determinants of their behavior in Anqiu County, China. The results show that the frequency of pesticide application by local farmers is high and that the improper disposal of pesticides after use is common in the study area. Although most farmers felt that they were at some degree of risk when using pesticides, farmers were found to overuse pesticides in the study area. The probability of pesticide overuse significantly decreased with farmers’ risk perceptions, willingness to reduce pesticide use, better social relationships, and strict government monitoring. The perception of risk can thus be an important element in education and communication efforts.

## 1. Introduction

Pesticides have played an important role in the success of modern food production since the green revolution in the early 1970s [1,2]. However, the intensive use of pesticides has had adverse effects on the environment and human health, making it an important public health concern [3,4,5].

China has a long history of cultivation and is now becoming the largest consumer of pesticides in the world [6], with the amount of pesticide use in China dramatically increasing in recent years. The growth of pesticide use will continue as agricultural production becomes more intense to meet the growing demand for food [7]. Local governments are endeavoring to regulate pesticide use and increase farmers’ awareness of hazards. To develop efficient policy interventions, evaluation of the quantities of pesticides used by the rural population is urgent [8,9].

The misuse or overuse of pesticides carries high risks to farmers’ health and the environment in China [10]. Some highly toxic, persistent, and bioaccumulative pesticides such as the chlorinated pesticides have been completely banned since 1983, but some of these are still commercially available [11]. Some high levels of residues are still detected in soils and water [12]. The poisoning and suicide case from pesticides are reported frequently [13].

Addressing farmers’ overuse or misuse of pesticides is a major challenge faced by China [9]. Although some research has been conducted to evaluate farmers’ pesticide use in China, most of this research has focused on commercial farmers, in particular, commercial cotton producers [14,15,16]. Small-scale farmers, who are the main contributors to China’s agriculture and play a key role in securing the food supply [9,10,16], are usually resource-poor, risk averse and heavy pesticide users [17]. However, empirical evidence regarding small-scale farmers’ pesticide overuse and its determinants is generally limited in China. In this study, we seek to quantify the extent of pesticide overuse and identify the determinants of pesticide practices among farmers who cultivate small-scale farmland in Anqiu County, China. The possible effects of farmers’ knowledge and risk perceptions on their behavior with regard to pesticide use are also investigated. To the best of our knowledge, this is the first study to empirically investigate the potential link between risk perceptions and overuse of pesticides among small-scale farmers in China. The findings of this study can aid the design of effective policies to address health problems and environmental issues due to pesticide overuse in China and other similar parts of the developing world.

The remainder of this study is organized as follows: Section 2 describes the study area, the research design and data collection. Section 3 reports the empirical results and discusses the findings. Conclusions and policy implications are presented in the final section.

## 2. Materials and Methods

### 2.1. Study Area

**Figure 1:**
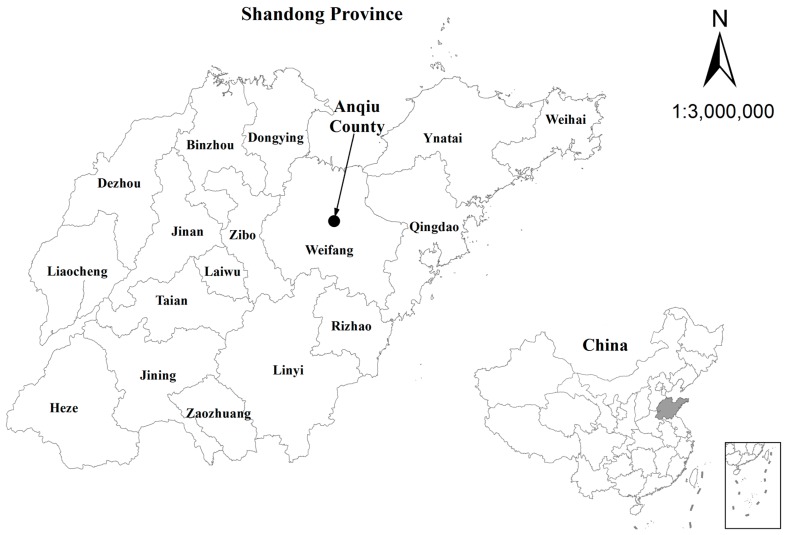
Location of the study area in China.

This study was conducted in Anqiu County, Shandong Province, China (Figure 1), located between 36°05′ and 36°38′ north latitude and 118°44′ to 119°27′ east longitude. It has a total land area of 1760 km^2^ and a population of 950,000. The average population density is 539 per km^2^.

The county has more than 866.7 km^2^ of arable land. Approximately 10 different crops are grown in the area. The principal crops are winter wheat, scallion and garlic, occupying 69% of the total arable land, followed by peanut, which occupies 13% of the total land area. The remaining 8% of the land is used mainly for fruit such as peaches and cherries. Irrigation is widely practiced, with groundwater as the main water source. The selection of the study area is based on the following criteria:

- Geographically, the county is located in the central part of the North China Plain. Agricultural production in this area plays an important role in ensuring food security for the country, and Anqiu County has now been identified as the national agricultural standardization demonstration city.
- The county has been adopting high input farming practices, including pesticides. Assessment of such intensive production practices has increasingly attracted the attention of academics, planners and decision-makers.
- The county can be seen as representative of the North China Plain in terms of biophysical, socio-economic and agricultural production conditions.

### 2.2. Survey Design

The survey questionnaire was developed and revised, using results from a series of focus group discussions and pre-test surveys. Focus group discussions were carried out among scientific experts on health and agriculture, government officials from the Agricultural Bureau and Sanitary Bureau of Anqiu County, and some local farmers. The purpose of the focus group discussions was to assess the suitability of the draft questionnaire and obtain opinions and key information on the knowledge, risk perceptions, attitudes and behaviors of local farmers regarding pesticide use. A series of pre-test surveys was issued to review the language and clarity of survey questions, to further identify and correct potential problems in the wording of the questionnaire, and to collect additional information regarding farmers’ knowledge and attitudes towards pesticide use. Based on the results of pre-test fieldwork, some modifications and clarifications were made.

The questionnaire used in the field consisted of three major sections. The first section was used to collect information on farmers’ knowledge and risk perceptions of pesticide use. The second section was used to collect information on respondents’ actual practices with regard to pesticide use. The final section was used to collect information on the socioeconomic characteristics of the respondents, including age, gender, formal education, income, years of farming experience, hectares of cultivated land, presence of children (under 16) in the family and health status.

### 2.3. Data Collection

Data collection took place between July and August 2016. To obtain information from the respondents, face-to-face interviews were conducted to encourage greater responsiveness. This survey method is expensive, but it can provide the highest response rates and is better suited to collecting complex information [8]. Professional interviewers were trained to effectively conduct face-to-face interviews.

Respondents were selected, based on random sampling, from eight towns of Anqiu County, using lists of inhabitants obtained from local village officials. A respondent was the individual who did the most farm work and used pesticides in his/her household. Participation was entirely voluntary, and potential respondents were free to refuse to participate without providing a justification. On average, it took about 30 min for respondents to finish the questionnaire survey. Each respondent received a token gift (shampoo, soap, toothpaste or towel), valued at US $1.53, as economic compensation. A total of 660 farmers were approached, and 630 participated in our study. The overall response rate (successful interviews completed) was 95%. The survey data from the 630 fully completed questionnaires were encoded and entered into Excel spread sheets and were then verified prior to analysis. The SPSS 20.0 (SPSS Inc., Chicago, IL, USA) and Stata/SE 13.0 (StataCorp LP, College Station, TX, USA) software were used in the data analysis.

## 3. Empirical Results and Discussion

### 3.1. Characteristics of Respondents

Table 1 reports descriptive statistics for the main socioeconomic characteristics of the sample. The majority of the respondents were male (*n* = 452, 71.74%), which is consistent with the actual situation in Anqiu, where men perform most agricultural activities and appear to take greater responsibility for purchasing and spraying pesticides. Farmers’ average age was 52.73 (±10.03) years. All respondents were married. Our random sample brought about a rather representative distribution of education levels: 7.62% of the respondents were illiterate, 18.89% had a primary school education, 56.98% had a middle school education, 16.03% had a senior high-school education, and only a few (0.48%) had a university degree. The mean household size of the sample was approximately 3.87, with a mean of 0.63 persons under 16 years of age. The average household income was approximately US $557/month. The farmers interviewed were typically small holders, with farm sizes averaging 0.60 hectares/household, and 89.84% of farmers’ land holdings were below 1.0 hectare. Our results show that most indicators of respondents’ socioeconomic characteristics are consistent with the average values for the whole country [15,16]. Thus, it can be concluded that our sample is representative.

### 3.2. Knowledge of Farmers Regarding Pesticide Use

Nearly all farmers interviewed (98%) believed that it is important to use or apply pesticides in a correct and scientific way. This is a welcome finding from the perspective of reducing the hazards of pesticides. When asked what problem they perceived as most important, approximately 42% of the respondents thought that how to use pesticides in a safe way was the most important problem, and 30% felt that the human health effects of pesticide use was most important.

Respondents were asked their opinions of several knowledge statements. Likert scaling, which is the most widely used psychometric scale in survey research, was applied in this study. Answer categories for these statements were based on a five-category Likert scale, going from 1 (strongly disagree) to 5 (strongly agree). The survey results are presented in Table 2.

Most respondents agreed or strongly agreed that pesticide use in food production reduces food safety. The respondents were well aware that pesticides are harmful to the environment and human health. The majority of the sample (over 80%) agreed or strongly agreed that pesticide use adversely affects human health or the environment. Only a few respondents agreed that increased pesticide use would be more effective in pest control. With regard to respondents’ perceptions of their personal health risks, most of the farmers thought using pesticides would adversely affect their own health. More than two-thirds of the sample claimed that they knew the interval between two sprays.

Most of the respondents (76%) stated that they had read or heard about illnesses caused by pesticides. More than half of the sample (66%) believed that illnesses caused by pesticide exposure can be fatal. Approximately half of the sample (50.32%) claimed that they had attended some type of training on how to use pesticides properly.

Table 3 reports on farmers’ main sources of information regarding pest control and pesticide application. It can be seen that the most important information source regarding pest control and how to apply pesticides is oral communication with other farmers, followed by pesticide retailers. Only 10.95% of the sample reported that they had acquired information on pest control and pesticide application from government extension services. Ineffective extension services are considered a key factor leading to the overuse of pesticides in developing countries [18]. Very few farmers learned about pesticides via media, e.g., the internet, television, books or newspapers.

### 3.3. Risk Perceptions of Farmers Regarding Pesticide Use

Respondents were asked to estimate the risk of pesticide use on their own health in terms of five proposed categories (Table 4). The findings are as follows: 15.87% of the sample reported an extremely high level of risk, 33.49% believed the risk to be large, 25.40% thought the risk to be medium or moderate, 23.33% reported a low level of risk, and 1.9% believed that there was no risk at all when using pesticides.

Thirty-eight percent of respondents reported having felt sick in the previous year after routine applications of pesticides. Approximately 23% of the sample stated that family members felt sick because of pesticide application. The most common pesticide poisoning symptoms reported by the interviewees were headache, nausea and vomiting. Kishi et al. [19] reported that farmers assume that pesticide poisoning symptoms were normal, so they could get used to them. This may explain why few farmers in this study reported symptoms of ill health after spraying.

### 3.4. Pesticide Use Practices of Farmers

The survey results show that respondents spent approximately CNY 1382 (US $213) on pesticides in 2015 and on average used approximately 19 kg of pesticides during the 2015 season. When asked where they purchased pesticide, the majority of respondents (90.14%) said they bought pesticides from special pesticide retail stores.

**Figure 2:**
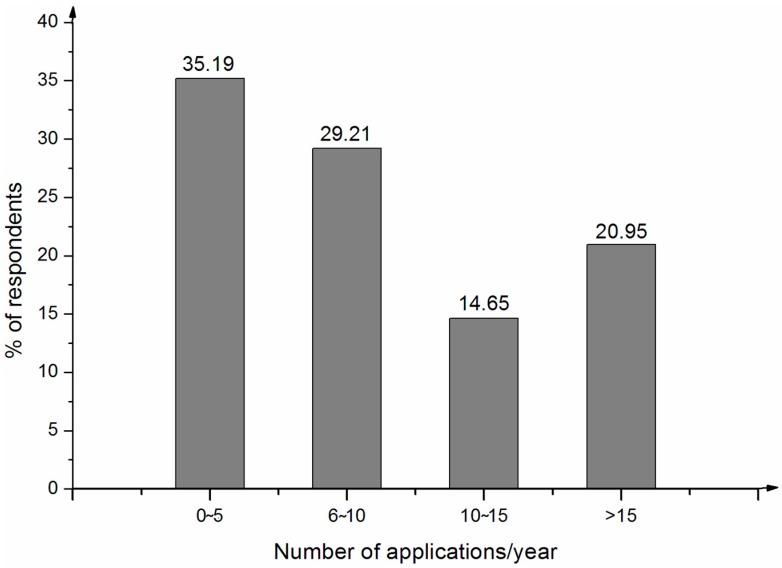
Number of pesticide applications.

Local farmers made wide use of knapsack sprayers—equipment that is relatively cheap, easily available and easy to operate and maintain. The average frequency of pesticide application for the sample was approximately eleven times per growing season. This finding is comparable to farmers in Pakistan, where approximately average number of pesticide applications per growing season is 10 or 11 [8]. Approximately 35% of the respondents applied pesticides up to 10 times or more. Approximately 21% of the farmers reported applying pesticides 15 times or more per year (Figure 2). Thus, the frequency of pesticide application by local farmers was high. Such heavy use of pesticides may result in frequent contact with pesticides, which can lead to significant health problems and possible air, soil and water pollution. Such heavy use of pesticides could occur because most local farmers were not receiving agricultural extension services. Sun et al. [20] conclude that inadequate agricultural extension services is the most important external factor in the overuse of chemical inputs, including pesticides.

Methods of storing and disposing of pesticide containers are also critical points of intervention that can be used to enhance awareness of safety during the application of pesticides [21]. Our results show that the majority of the sample (89.91%) stored pesticides in their homes, but only 23.13% of the farmers said they had stored pesticides in specific storerooms. Pesticides stored in the home can easily contaminate drinking water and food and can threaten the health of children [22].

The improper disposal of pesticide containers after use is a common practice in the study area. Nearly half of the farmers (47.14%) discarded empty containers near the fields where they prepared the pesticides. This improper disposal of pesticide containers after use can easily lead to contamination of agricultural soil and water. Only 20.63% of the sample claimed that they disposed of containers as garbage. Disposal of containers by burning or burying them in fields was reported, but the proportion of containers disposed of via these methods was less than 25%.

### 3.5. Farmers’ Overuse Behaviors

Our survey respondents were asked, among other questions, “Compared to the dosage that is recommended by the manual, how much do you usually use?” Our results show that approximately 69.21% of the sample applied the pesticide based on the manual or label on the pesticide container. However, approximately 17.78% of the sample used a higher dosage than recommended on the label, and 12.54% of the sample applied the pesticide based on their own experience.

To identify the factors affecting farmers’ decisions regarding pesticide overuse, we performed regressions, using the binary logistic model [23,24]. The specification or reduced form of the empirical model estimated is as follows:

(1) $$Y_{i}=\alpha +\beta_{i}\sum^{\text{​}}X_{i}+\varepsilon_{i}$$

where $Y_{i}$ is a dichotomous dependent variable (farmer using pesticide more than the amount recommended on the label, specified as yes = 1, 0 = otherwise); *α* is the *Y*-intercept; *β_i_* is the set of coefficients to be estimated; *X* is the set of explanatory variables hypothesized, based on theory and related empirical work, to influence farmers’ decisions; and *ε* is an error term.

Using Equation (1), we run the binary logistic regression model. The regression results are presented in Table 5. Most explanatory variables take the expected signs and are statistically significant at the 10% or lower levels. The results of a *chi*-square test show that the likelihood ratio statistics are highly significant (*p* < 0.001), suggesting that the explanatory power of the regression model is strong.

The regression results show that the coefficient for farmers’ risk perception is negative and significant (see Table 5), indicating that farmers who perceive a higher health risk of pesticide use are less likely to overuse pesticide. This result is consistent with the finding in the literature that farmers’ perceptions of pesticide risk influence their behavior towards pesticide use [15,25,26]. Earlier empirical studies also find that perceptions of low health risk from pesticide use are positively correlated with overuse [27,28].

The coefficient on farmers’ willingness to reduce pesticide use is negative and significant, indicating that if a farmer is willing to reduce pesticide use in the future, she or he will have a lower probability of overusing pesticides. The regression results also indicate that reading the label on the pesticide container negatively affects pesticide overuse. This finding is as expected and understandable. If farmers read the label on the pesticide container before they use the pesticide, they would be more likely to use the dosage recommended on the label, reducing the probability of pesticide overuse.

The variable “Farmyears” is positive and significant, indicating that more experienced farmers are more likely to overuse pesticide. Older farmers would thus appear to use more pesticides than younger farmers, as farming experience tends to be directly related to age. Older farmers usually have lower levels of education and perceive lower risks and greater benefits from using pesticides [25]. Therefore, farmers who have used pesticides for a long time are more likely to overuse pesticides. Fan et al. [29] argue that older farmers have more difficulty than younger farmers following instructions for pesticide use, leading to inappropriate behaviors.

As expected, the coefficient on “Governance” is positive and significant, indicating that if a farmer believes that the local government strictly supervises pesticide use, he will be less likely to use pesticides more than recommended. The same result has been reported in studies in African countries [30].

Interestingly, the regression results show that farmers’ social relationships negatively affect the probability of pesticide overuse. Specifically, if respondents have better relationships with other villagers, they are less likely to overuse pesticides. Thus, there is a need to build farmers’ social capital, enabling them to redesign agro-ecosystems to be more productive while lowering the use of pesticides.

Our results also show that farmers’ income negatively and significantly affects the probability of pesticide overuse. This finding is not surprising, as rich farmers are typically more concerned than poorer farmers about health hazards associated with pesticides, which may reduce pesticide overuse.

## 4. Conclusions

China is the world’s largest user of pesticides, and their use is growing rapidly [9]. Unsafe use and misuse of pesticides in China are major threats to farmers’ health and the environment. Information regarding farmers’ risk perceptions and behavior with regard to pesticides is a prerequisite for any policy intervention initiatives [8]. The purpose of this study is therefore to investigate small-scale farmers’ knowledge, risk perceptions, and practices regarding pesticide use in Anqiu County, China. The findings of this study will provide data for ongoing efforts in the region to promote upstream policy interventions for reduction of hazardous pesticide exposure of vulnerable small-scale farmers [31].

The results of this study indicate that local farmers have some knowledge of the adverse effects of pesticide use on human health and the environment. The frequency of pesticide application by local farmers is high. Improper disposal of pesticides after use was found to be common in the study area. Most respondents felt that they were at some degree of risk when using pesticides. Almost half of the respondents viewed their health risk from pesticide use as high or extremely high.

Oral communications with other farmers and pesticide retailers are the two most important information sources for local farmers regarding pesticides and the amounts of pesticides to apply. Current training on pesticide use and alternatives are inadequate, and there is a need to launch education or training programs to educate farmers, particularly the elderly, to enhance their knowledge of pesticides and how to properly use pesticides. Pesticide retailers must also be educated, trained and supervised to improve their ability to provide clear and standard information to farmers. Most local farmers did not receive any agricultural extension services from local governments. Because extension services can offer appropriate advice on pesticide applications [31], there is an urgent need that the capacity of the extension system be strengthened to enable it to more effectively provide farmers with information on pesticide use in the study area.

Local farmers were found to overuse pesticides in the study area, but such overuse was not found to be as common as reported in previous studies [32]. The probability of pesticide overuse significantly decreased with farmers’ risk perceptions, strict government supervision and reading by farmers of labels on pesticide containers. Therefore, perception of risk can be an important element in developing effective campaigns of education and communication. In addition, information regarding the environmental and health hazards induced by pesticide overuse should be widely disseminated. Finally, to reduce the probability of pesticide overuse, the effective legislation and monitoring of farmers’ pesticide use and application methods could be strengthened.

## Figures and Tables

**Table 1:** Main socioeconomic characteristics of all the sample.

Variable	Mean	Standard Deviation	Min	Max
Male respondent	0.71	0.45	0	1
Age of the respondent	52.73	10.03	18	86
Marry status	1.00	0.00	1	1
Total household members	3.82	1.50	1	10
Total household income (CNY/month)	3621	3057	250	17,500
Total farm area (ha/household)	0.60	1.02	0.31	20

**Table 2:** Respondents’ perceptions on pesticide use.

Statements	5	4	3	2	1
The use of pesticides in food production reduces the safety of food.	42.77	40.70	6.84	7.31	2.38
Pesticide use will have adverse effects on the environment.	45.87	37.78	6.19	7.30	2.86
Pesticide use have harmful effects on human health.	45.71	43.17	5.24	4.76	1.11
A larger amount of pesticide use will have better effects on pest control.	12.86	12.54	11.43	37.30	25.87
Pesticide use will have negative effects on my health.	40.63	41.43	9.87	5.71	2.38
I know the pesticide safety interval period.	12.38	59.52	10.48	16.67	0.95

Note: 5 = strongly agree; 4 = agree; 3 = neutral; 2 = disagree; 1 = strongly disagree.

**Table 3:** Information source of pest control and pesticide use.

Source	Percent (%)
Communication between other farmers	45.24
Government extension services	10.95
Pesticide retailers	34.44
Media	9.35

**Table 4:** Perceptions of pesticide risk on farmers’ health.

Risk Perception	No. Farmers	Percentage
Extremely high level of risk	100	15.87
Large level of risk	211	33.49
Medium level of risk	160	25.40
Some small level of risk	147	23.33
No risk at all	12	1.90

**Table 5:** Factors affecting farmers’ pesticide overuse.

Variable	Description		Estimate	Standard Error	*p*-Value
Risk	Risk perception (1 = no risk, 2 = small risk, 3 = medium risk, 4 = high risk and 5 = extremely high risk)		−0.10 **	0.05	0.051
Reduction	Willingness to reduce pesticide use (1 = yes; 0 = otherwise)		−0.29 ***	0.11	0.010
Read	Reading labels on pesticide containers (1 = yes, 0 = no)		−0.73 ***	0.16	0.000
Governance	Local government having a strict supervision on pesticide use (1 = extremely strict; 2 = strict; 3 = moderate; 4 = loose; 5 = extremely loose)		0.10 *	0.06	0.088
Farmyears	Years of farming experience		0.01 **	0.00	0.02
Income	Monthly income of the respondent (CNY 1000)		−0.07 *	0.04	0.086
Relationship	Better relationships with other villagers (1 = extremely bad; 2 = bad; 3 = moderate; 4 = good; 5 = extremely good)		−0.19 **	0.08	0.024
Knowledge	Knowledge score		0.06	0.05	0.191
Constant			0.61	0.50	0.224
Summary statistics					
Log likelihood		−353.34			
LR *chi*-squared [9]		66.36			
Prob > *chi*^2^		0.000			
Observations		630			

*** Significant at 1% level; ** Significant at 5% level; * Significant at 10% level.
